# The development of simple survival prediction models for blunt trauma victims treated at Asian emergency centers

**DOI:** 10.1186/1757-7241-20-9

**Published:** 2012-02-02

**Authors:** Akio Kimura, Shinji Nakahara, Witaya Chadbunchachai

**Affiliations:** 1 Department of Emergency Medicine & Critical Care, National Center for Global Health and Medicine, Hospital, Shinjuku, Tokyo, 162-8655, Japan; 2 Department of Preventive Medicine, St. Marianna University School of Medicine, Miyamae, Kawasaki, Kanagawa, 216-8511, Japan; 3 Trauma and Critical Care Center, Khon Kaen Regional Hospital, Srichan Road, Khon Kaen, 40000, Thailand

## Abstract

**Background:**

For real-time assessment of the probability of survival (Ps) of blunt trauma victims at emergency centers, this study aimed to establish regression models for estimating Ps using simplified coefficients.

**Methods:**

The data of 10,210 blunt trauma patients not missing both the binary outcome data about survival and the data necessary for Ps calculation by The Trauma and Injury Severity Score (TRISS) method were extracted from the Japan Trauma Data Bank (2004-2007) and analyzed. Half (5,113) of the data was allocated to a derivation data set, with the other half (5,097) allocated to a validation data set. The data of 6,407 blunt trauma victims from the trauma registry of Khon Kaen Regional Hospital in Thailand were analyzed for validation. The logistic regression models included age, the Injury Severity Score (ISS), the Glasgow Coma Scale score (GCS), systolic blood pressure (SBP), respiratory rate (RR), and their coded values (cAGE, 0-1; cISS, 0-4; cSBP, 0-4; cGCS, 0-4; cRR, 0-4) as predictor variables. The coefficients were simplified by rounding off after the decimal point or choosing 0.5 if the coefficients varied across 0.5. The area under the receiver-operating characteristic curve (AUROCC) was calculated for each model to measure discriminant ability.

**Results:**

A group of formulas (log (Ps/1-Ps) = logit (Ps) = -9 + cISS - cAGE + cSBP + cGCS + cRR/2, where -9 becomes -7 if the predictor variable of cRR or cISS is missing) was developed. Using these formulas, the AUROCCs were between 0.950 and 0.964. When these models were applied to the Khon Kean data, their AUROCCs were greater than 0.91. Conclusion: These equations allow physicians to perform real-time assessments of survival by easy mental calculations at Asian emergency centers, which are overcrowded with blunt injury victims of traffic accidents.

## Background

The Trauma and Injury Severity Score (TRISS) [1,2] is a standard method for estimating survival and is often used to evaluate the quality of trauma care. However, it requires the Injury Severity Score (ISS) [3], the Revised Trauma Score (RTS) [4] calculated based on the Glasgow Coma Scale score (GCS), the systolic blood pressure (SBP), the respiratory rate (RR), and the categorically coded value of age (cAGE). The formulas are:

$$\begin{array}{c} \text{Probability}\;\text{of}\;\text{survival}\;\text{(Ps)}=\text{1/(1}+{\text{e}}^{-\text{b}}\text{),}\\ \text{where}\;\text{b}=-\text{0}\text{.4499}-\text{0}\text{.0835*ISS}+\text{0}\text{.8085*RTS}-\text{1}\text{.743*cAGE}\;\text{[2]}\\ \text{RTS}=0.9368*\text{cGCS}+\text{0}\text{.7326*cSBP}+\text{0}\text{.2908*cRR}\;\left[4\right] \end{array}$$

Collecting all of this information and performing such complex calculations are not feasible in the clinical setting at emergency centers. For clinicians, especially emergency physicians, it is hoped that a way to predict survival of trauma victims more easily without a significant decrease in accuracy could be developed.

This study aimed to establish regression models for quick assessment of Ps for blunt trauma (BT) victims based on simplified coefficients that could be used even when the variable of RR or the variable of ISS is missing. The former is frequently missing in the trauma registry data of Japan [5], and the latter is rarely determined during the early stage of trauma management in most cases.

## Methods

### Study design, population, and settings

A retrospective observational study was conducted to create Ps prediction models with simple coefficients for BT victims in Japan and Thailand.

Once approval was obtained from the trauma registry committee of the Japanese Association for the Surgery of Trauma, deidentified, anonymous data from the Japan Trauma Data Bank (JTDB), with which 144 Japanese hospitals have been involved since 2004, were used [6].

Data (10,210) that were not missing both outcome data about survival and the predictors necessary for Ps calculation by the TRISS method were collected from BT patients (17,564) registered in the JTDB from 2004 to 2007. Half (5,113) of the data was randomly allocated to a derivation data set, with the remaining half (5,097) allocated to a validation data set.

For international validation, with the permission of the hospital, the proposed equations were applied to 6,409 of 6,667 BT patients injured in the Khon Kaen District in Thailand between January 2005 and December 2008 and collected in the Khon Kaen Regional Hospital Trauma Registry, where data have been collected since 1997. This hospital is one of the World Health Organization (WHO) collaborating centers for injury prevention and safety promotion. The data of two patients were excluded because they were erroneous.

The independent variable was survival (survival = 1; non-survival = 0). Age, the ISS, the GCS, SBP, RR, and their coded values (cAGE, cISS, cSBP, cGCS, cRR), defined in Table 1, were used as predictor variables. The GCS, SBP, RR, and age were coded according to the RTS [4] and the TRISS method [1,2]. For ISS categorization to cISS, recursive partitioning, which is an exploratory technique to split a dataset into increasingly homogeneous subgroups having the greatest difference between the groups at each stage, was conducted with reference to previous literature [7,8].

**Table 1:** Coded Values

Coded value	Glasgow coma scale score	Systolic blood pressure (mmHg)	Respiratory rate (/min)	Age (years)	Injury Severity Score
**4**	13-15	> 89	10-29		< 16
**3**	9-12	76-89	> 29		16-24
**2**	6-8	50-75	6-9		25-40
**1**	4-5	1-49	1-5	≥ 55	41-65
**0**	< 4	No pulse	0	0-54	> 65

### Analyses

Logistic regression analyses were applied to establish the models. The maximum likelihood estimation was used as the method of coefficient estimation for each model.

For model selection, Akaike's Information Criterion (AIC) [9], - 2log (maximum likelihood) +2 (number of adjusted parameters), was used. The model having the lower AIC is considered to be better fitting. The area under the receiver-operating characteristic curve (AUROCC), which distinguishes between survival and non-survival, and varies between 0.5 (= no discrimination) and 1 (= perfect discrimination), of each model was also measured.

The coefficients were simplified by rounding off after the decimal point or choosing 0.5 if it was nearer to the coefficients than 0 or 1.

The JMP 9.0 (SAS Institute Inc.) and SAS 9.1 (SAS Institute Inc.) software packages were used for statistical analyses.

The protocol of the present study was approved by the Ethics Committee of the National Center for Global Health and Medicine.

## Results

Distributions of predictor variables and the proportion of survivors of each data set are shown in Table 2. The characteristics were substantially different between the Khon Kaen data and the JTDB data, in which both the derivation data and the validation data were similar.

**Table 2:** Distribution of Variables

		Derivation Data	Validation Data	Khon Kaen Data
Number		5113	5097	6407
cAGE	0	58.0%	58.5%	87.6%
	1	42.0%	41.5%	12.4%
RTS		7.8 [6.9, 7.8]	7.8 [6.9, 7.8]	7.8 [7.8, 7.8]
cSBP	4	85.0%	85.3%	95.9%
	3	3.2%	3.4%	1.6%
	2	2.6%	2.6%	1.1%
	1	1.3%	1.1%	0.2%
	0	7.9%	7.6%	1.2%
cGCS	4	72.4%	73.4%	89.4%
	3	7.5%	7.0%	3.0%
	2	6.1%	5.9%	4.6%
	1	2.6%	2.6%	1.1%
	0	11.4%	11.1%	1.9%
cRR	4	76.0%	76.8%	92.1%
	3	15.0%	14.8%	0.2%
	2	0.4%	0.4%	0.02%
	1	0.2%	0.1%	0.2%
	0	8.4%	7.9%	7.5%
ISS		17.6 ± 14.2	17.4 ± 14.0	9.5 ± 10.1
cISS	4	51.1%	51.0%	83.2%
	3	21.2%	22.3%	6.9%
	2	20.4%	19.7%	7.4%
	1	5.3%	5.2%	2.3%
	0	2.0%	1.8%	0.2%
Survival		82.1%	83.1%	95.9%

cAGE: coded value of age, RTS: the Revised Trauma Score shown by median [IQR],

cBP: coded value of systolic blood pressure, cGCS: coded value of the Glasgow Coma Scale score,

cRR: coded value of respiratory rate, ISS: the Injury Severity Score shown by mean ± standard deviation,

cISS: coded value of the Injury Severity Score

The AIC and the AUROCC of each model are shown in Table 3. Over the set of models, the model with cISS, cAGE, cSBP, cGCS, and cRR as predictor variables showed the smallest AIC (1732), which was even smaller than the AIC for the model using ISS, RTS, and cAGE. Initially, six models that used only coded values and had lower AICs than that of the original TRISS model (1988) were selected for simplification. Then, the two models that had a higher AUROCC than the TRISS model and one model that does not require cISS were selected.

**Table 3:** Akaike's Information Criterion (AIC) and Discriminant Abilities for Each Model

Predictor variables used for each regression model	AIC	AUROCC
*ISS, RTS, cAGE (original TRISS)*	1988	0.9627
ISS, RTS, cAGE	**1788**	**0.9637**
ISS, cAGE, cSBP, cGCS, cRR	**1791**	**0.9637**
**cISS, cAGE, cSBP, cGCS, cRR**	**1732**	**0.9648**
**cISS, cAGE, cSBP, cGCS**	**1748**	**0.9649**
cISS, cAGE, cGCS, cRR	**1819**	0.9609
cISS, cSBP, cGCS, cRR	**1846**	0.9561
cISS, cSBP, cGCS,	**1854**	0.9562
**cAGE, cSBP, cGCS, cRR**	**1987**	0.9503
cAGE, cSBP, cGCS	2000	0.9465
cISS, cAGE, cGCS	2001	0.9547
cISS, cAGE, cSBP, cRR	2017	0.9481
cISS, cAGE, cBP	2024	0.9433

AUROCC: The area under the receiver-operating characteristic curve,

cAGE: coded value of age, RTS: the Revised Trauma Score, cBP: coded value of systolic blood pressure,

cGCS: coded value of the Glasgow Coma Scale score, cRR: coded value of respiratory rate,

ISS: the Injury Severity Score, cISS: coded value of the Injury Severity Score

The estimated coefficients of the logistic regression models derived from the training data are shown with the original TRISS coefficients in Table 4. Each coefficient of cSBP, cGCS, and cRR on the TRISS line of the table was obtained from each coefficient of the RTS (0.7326, 0.9368 and 0.2908, respectively) multiplied by the coefficient of the RTS (0.8085) of the TRISS method using the 1990 version of AIS [2]. All estimated coefficients were significant.

**Table 4:** Coefficients of Logistic Regression Models

Models with predictor variables	Intercept	β(c)ISS	βRTS	βcAGE	βcGCS	βcBP	βcRR
*Original TRISS *	-0.4499	-0.0835	0.8085	-1.743	0.7574	0.5923	0.2351
ISS, RTS, cAGE	-1.7162* (0.279) [37.8]	-0.0675* (0.005) [181]	0.9301* (0.0368) [639]	-1.439* (0.137) [111]	*	*	*
ISS, cAGE, cSBP, cGCS, cRR	-1.8646 (0.340) [30.2]	-0.0678* (0.0050) [181]	*	**-**1.452* (0.137) [112]	0.846* (0.047) [328]	0.670* (0.077) [75.8]	0.346* (0.090) [14.9]
**cISS, cAGE, cSBP, cGCS, cRR**	-6.281* (0.335) [351]	**1**.**0**58* (0.070) [227]	*	**-1**.**4**04* (0.137) [104]	**0.7**77* (0.047) [267]	**0.7**18* (0.077) [87.5]	**0.3**70* (0.090) [17.0]
**cISS, cAGE, cSBP, cGCS**	-5.734* (0.283) [410]	**1**.**0**38* (0.069) [225]	*	**-1**.**3**48* (0.136) [98.8]	**0.8**41* (0.045) [345]	**0.8**89* (0.063) [202]	x
**cAGE, cSBP, cGCS, cRR**	-4.663* (0.357) [170]	x	*	**-1**.**3**28* (0.129) [105]	**1.0**25* (0.044) [540]	**0.8**43* (0.077) [122]	**0.3**49* (0.094) [13.7]

Βx: regression coefficients, *: p < 0.001, (standard error), [likelihood ratio chi-square value],

PVs: predictor variables, cAGE: coded value of age, RTS: the Revised Trauma Score,

cBP: coded value of systolic blood pressure, cGCS: coded value of the Glasgow Coma Scale score,

cRR: coded value of respiratory rate, ISS: the Injury Severity Score,

cISS: coded value of the Injury Severity Score

The coefficients of cISS, cAGE, cSBP, and cGCS in Table 4 were rounded off after the decimal point, and the coefficient of cRR was regarded as 0.5.

The three developed models were as follows:

$$\begin{array}{c} \text{logit (Ps)}=\text{intercept}+\beta =-9+\text{cISS}-\text{cAGE}+\;\text{cSBP}+\text{cGCS}+\text{cRR/2,}\\ \text{logit (Ps)}=\text{intercept}+\beta =-7+\text{cISS}-\text{cAGE}+\;\text{cSBP}+\text{cGCS},\\ \text{logit (Ps)}=\text{intercept}+\beta =-7-\text{cAGE}+\;\text{cSBP}+\text{cGCS}+\text{cRR/2,}\\ \text{,}\;\text{where}\;\text{logit}\;\text{(Ps)}=\text{log}\;\left(\text{Ps/1}-\text{Ps}\right) \end{array}$$

As for the intercept of each model, the nearest integer (-7 or -9) to -**β**, where actual survival proportions just crossed 50% in the derivation data set, namely logit (Ps = 0.5) = 0, was chosen (Table 5).

**Table 5:** β value and Actual Survival Percentage in the Derivation Data

β	cISS-cAGE+cSBP+cGCS+cRR/2 Survival (%)	cISS-cAGE+cSBP+cGCS Survival (%)	-cAGE+cSBP+cGCS+cRR/2 Survival (%)
-1	0.0	0.0	0.0
0	0.0	0.0	0.0
1	0.0	0.0	0.0
2	0.0	0.0	0.0
3	1.7	11.8	30.0
4	14.0	19.7	28.0
5	30.0	26.8	33.8
6	32.4	40.3	47.6
		
**7**	28.8	**51.5**	**66.5**
8	38.3	75.3	82.7
			
**9**	**58.3**	88.5	96.5
10	78.7	96.7	99.5
11	91.4	98.6	
12	96.1	99.9	
13	98.5		
14	99.9		

If only one variable cannot be obtained, then a zero value is given for the missing predictor variable in each equation.

Actual survivals just crossed 50% around the nearest integer value of **β **(7 or 9)

cAGE: coded value of age, cBP: coded value of systolic blood pressure, cGCS: coded value of the Glasgow Coma Scale score, cRR: coded value of respiratory rate, cISS: coded value of the Injury Severity Score

For all models, including the models with missing variables, the AUROCCs were greater than 0.95 (Table 6). The same results were also shown for the Japanese validation data.

**Table 6:** Proposed Regression Models with Simplified Coefficients

Logit (Ps) of each model	AUROCC JTDB derivation data	AUROCC JTDB validation data	AUROCC Khon Kaen registry data
**-9 + cISS - cAGE + cSBP + cGCS + cRR/2**	0.9635	0.9640	0.9619
**-7 + cISS - cAGE + cSBP + cGCS**	0.9633	0.9622	0.9601
**-7 + cAGE + cSBP + cGCS + cRR/2**	0.9503	0.9524	0.9115

AUROCC: The area under the receiver-operating characteristic curve,

cAGE: coded value of age, cBP: coded value of systolic blood pressure,

cGCS: coded value of the Glasgow Coma Scale score, cRR: coded value of respiratory rate,

cISS: coded value of the Injury Severity Score

The AUROCCs of each model, applied to the data of the Khon Kaen Trauma Center in Thailand, are also shown in Table 6. The two models with cISS, cAGE, cSBP, cGCS as predictor variables showed AUROCCs greater than 0.96, almost the same as that of the TRISS model. For the model without cISS, the AUROCC was even greater than 0.91.

## Discussion

The TRISS [1,2] method is the most popular method of survival estimation. However, it is not a suitable tool for quick assessment of survival probability in the clinical setting, because it requires complicated calculations using the ISS, for which precise coding of the Abbreviated Injury Scale (AIS) [10] is required, and the RTS, which also has complex coefficients for cGCS, cSBP, and cRR. Therefore, we tried to simplify Ps prediction without a significant decrease in accuracy for clinical rather than for administrative uses. Without substantial loss in the AUROCC compared with the original TRISS, the present study showed that logit (Ps) can be obtained even with a marked simplification of variables and intercepts (Table 6), sufficient to enable its mental calculation. In any case where logit (Ps) is greater than 0, by easy mental calculation it provides for quick determination as to whether the Ps is greater than 0.5, which is considered the lower limit for decision making about unexpected trauma death.

In addition to a recent report [11], the present study also directly used cSBP, cGCS, and cRR as predictor variables instead of the RTS. The ISS was coded based on our recent paper [7], and predictive models using the cISS were successfully constructed. Some of these models showed even smaller AICs or larger AUROCCs than those of the models using the ISS (Table 3). An important benefit of using the cISS instead of the ISS is that we can determine the cISS even without the information of the third most severe AIS score, which is sometimes lacking in physicians' records, as shown in Table 7 which was constructed with reference to Copes et al. [7]. This shows that, by dividing all variables into categories with adequate intervals, it is possible to perform valid coding in cases where only an approximate value, not the exact value of ISS, is known.

**Table 7:** Relationship between Coded ISS and Most Severe Abbreviated Injury Scale (AIS)

Coded ISS	ISS Interval	Most severe AIS/2^nd ^most severe AIS Included
4	< 16	**3**
3	16-24	**4**
2	25-40	**5 **or **4**/3
1	41-65	Two **5 **or **5**/4
0	> 65	Two **5**/4 or Three **5 **or **6**

ISS: the Injury Severity Score

Moreover, it was also shown that even if the cISS is undetermined, Ps calculation is nevertheless possible using just the age and vital sign factors, with only a slight decline in the AUROCC. This means that it is possible to predict Ps with high accuracy even for initial assessment at emergency centers in cases of undetermined anatomical severity. If a quick reference chart of Ps like Table 8 is prepared and kept in the pocket of physicians with Tables 1 and 7, Ps can be predicted without a computer. It can be used for hospital triage during initial management in case of a large number of BT victims, especially in multiple traffic or railroad accidents.

**Table 8:** Probability of Survival (Ps) Chart

b1~3	< -3	-2.5	-2	-1.5	-1	-0.5	0	0.5	1	1.5	2	2.5	> 3
Ps	< 0.05	0.08	0.12	0.18	0.27	0.38	0.5	0.62	0.73	0.82	0.88	0.92	0.95

Ps = 1/(1 + e^-b1~3^)

**b1 = **- **9 **+ cISS - cAGE + cSBP + cGCS + cRR/2

**b2 = **- **7 **+ cISS - cAGE + cSBP + cGCS

**b3 = **- **7 **- cAGE + cSBP + cGCS + cRR/2

In Japan, RR information is frequently deficient [5], but with the regression equation presented in this study, even without information on cRR, only a slight decline in AUROCC is seen. As shown in Table 4 cRR had the lowest chi-square value in each model. This indicates that, based on their experience, Japanese surgeons or emergency physicians appear to have realized that RR is a less important indicator for survival in BT patients. From the results of Table 6 RR also seems to be unimportant in Thailand, because the models without cRR showed only a slight decline in the AUROCC. The deficiency that most increases the AIC and reduces the AUROCC is cGCS (Table 3), which had the highest chi-square value in each model (Table 4). Thus, the level of consciousness is the most important factor at the time of survival prediction. The importance of information on consciousness level was also proven in a different way in our recent paper [12].

The present study had a few limitations that might have biased the results. Because of missing data related to survival and the predictors, the Ps calculation of the TRISS could be done in only 58% of 17,564 BT patients registered in the JTDB. Tohira et al. [13] pointed out that significant differences existed in age, RTS, and ISS between outcome-missing data and non-outcome-missing data, and that selection bias may exist in research outputs gained from the extracted data from the JTDB by excluding patients with missing outcomes and the TRISS predictors. Thus, it seems to be of great worth to validate the developed models with the Khon Kaen trauma registry data, which are substantially different from the JTDB data in age, RTS, and ISS. At present, the simplified models in this study have been validated only with the Japan Trauma Registry data and the Khon Kaen Trauma Registry data in Thailand. If the results of the present study can be verified with other data from around the world, especially from middle-income countries where traffic injuries are rapidly increasing [14], it will be of greater use internationally.

## Conclusion

The proposed, simplified equations allow quick assessments of Ps by simple mental calculation, which should prove useful at Asian emergency centers overcrowded with traffic accident victims suffering from blunt trauma.

## Competing interests

The authors certify that none of the authors has any financial or other relationships that could lead to a conflict of interest.

## Authors' contributions

AK made substantial contributions to conception, acquisition of JTDB data, analysis and interpretation of data. SN was involved in drafting the manuscript or revising it critically for important intellectual content. WC made substantial contributions to acquisition of data from the Khon Kaen Regional Hospital Trauma Registry. All authors read and approved the final manuscript.
